# The impact of chronic widespread pain on health status and long-term health predictors: a general population cohort study

**DOI:** 10.1186/s12891-020-3039-5

**Published:** 2020-01-16

**Authors:** Charlotte Sylwander, Ingrid Larsson, Maria Andersson, Stefan Bergman

**Affiliations:** 1 Spenshult Research and Development Centre, Bäckagårdsvägen 47, SE-302 74 Halmstad, Sweden; 2 School of Health and Welfare, Halmstad University, Halmstad, Sweden; 3 Department of Clinical Sciences, Section of Rheumatology, Lund University, Lund, Sweden; 4 Primary Health Care Unit, Department of Public Health and Community Medicine, Institute of Medicine, The Sahlgrenska Academy, University of Gothenburg, Gothenburg, Sweden

**Keywords:** Chronic widespread pain, Gender differences, Health predictors, Health status

## Abstract

**Background:**

Chronic widespread pain (CWP) has a negative impact on health status, but results have varied regarding gender-related differences and reported health status. The aim was to study the impact of CWP on health status in women and men aged 35–54 years in a sample of the general population. The aim was further to investigate lifestyle-related predictors of better health status in those with CWP in a 12- and 21-year perspective.

**Method:**

A general population cohort study including 975 participants aged 35–54 years, with a 12- and 21-year follow-up. CWP was measured with a pain mannequin, and the questionnaire included questions on lifestyles factors with SF-36 for measurement of health status. Differences in health status were analysed with independent samples t*-*test and health predictors with logistic regression analysis.

**Results:**

The prevalence of CWP was higher in women at all time points, but health status was reduced in both women and men with CWP (*p* < 0.001) with no gender differences of clinical relevance. At the 12-year follow-up, a higher proportion of women than men had developed CWP (OR 2.04; CI 1.27–3.26), and at the 21-year follow-up, a higher proportion of men had recovered from CWP (OR 3.79; CI 1.00–14.33). In those reporting CWP at baseline, a better SF-36 health status (Physical Functioning, Vitality or Mental Health) at the 12-year follow-up was predicted by male gender, having personal support, being a former smoker, and having no sleeping problems. In the 21-year follow-up, predictors of better health were male gender, a weekly intake of alcohol, and having no sleeping problems.

**Conclusion:**

Women and men with CWP have the same worsening of health status, but men recover from CWP to a greater extent in the long-term. Being male, having social support, being a former smoker, and having no sleeping problems were associated with better health status in those with CWP.

## Background

Chronic pain is a common health problem [1] and the term refers to pain that persists or recurs for more than three months [2]. The prevalence of chronic pain in the general population ranges from 13 to 25% [3–5], being higher in women [3, 4, 6], and increasing with age [3, 5, 6]. Chronic pain is associated with poorer general health [6–8], and people with chronic pain show a higher level of depression, anxiety, helplessness, and dissatisfaction [8, 9]. Chronic pain has a negative impact on daily living and work activity [5, 8]. It affects the ability to do household chores, walk, exercise, drive a car, concentrate, sleep, and it also affect sexual relations, the possibility of attending social activities, and the ability to maintaining an independent lifestyle [5, 8].

Chronic widespread pain (CWP) is a more severe form of chronic pain, it is usually defined according to the American College of Rheumatology (ACR) criteria as pain lasting for a minimum of three months, present on both sides of the body, below and above the waist, and in the axial skeleton [10]. The average estimated prevalence of CWP ranges from 10 to 15% [11, 12], and CWP causes not only suffering for the individual but also costs to society, with a high frequency of sick leave [8, 13]. There are several risk factors for development of CWP—for example, older age [12, 13], female gender [12, 13], a lower level of education [14], smoking [15, 16], excessive body mass [16, 17], lack of social support [14], sleep disorders [18, 19], and anxiety and/or depression [16]. Reversely, male gender, younger age, higher socioeconomic status, having emotional support, and suitable sleeping habits are associated with a better health status in those with CWP [6].

Research on pain and gender has increased markedly since 1995 [20], but even so, little is known about the causes of the differences in prevalence and experiencing of pain [20–22]. Many attempts have been made to find answers, but with contradictory results. Biological factors such as hormones and neurochemistry [20, 23], psychosocial [23, 24], and methodological bias [22] are all part of the current explanations. One study showed that after an educational programme including definitions and pathophysiology of chronic pain, the difference in prevalence between genders was no longer statistical significant [25]. These results regarding unawareness are supported by Järemo et al. [9] arguing a need for a better understanding of CWP since results showed that patients who had constraining beliefs about their chronic pain also had a lower health status.

It is recognised that people with CWP have a substantially worse health status [6, 7, 9], but there have been few studies on possible differences between men and women. Most studies have only examined chronic pain, and these results have been contradictory. For example, one study found no gender differences of clinical relevance regarding health status [26], another study found that men with chronic pain had a lower self-reported quality of life than women with chronic pain [4], and a third found that such women reported having anxiety and depression more often than their male counterparts [25].

There have not been any gender differences found regarding the impact of chronic pains on daily activities, apart from the ability to perform daily chores where men reported less of an effect [5]. Furthermore, no gender differences have been found regarding how much the participants were bothered by pain [4]. Healthy lifestyle-related behaviour could reduce the risk of developing chronic pain and improve health status when suffering from chronic pain [27], but little is known about gender differences regarding the impact of CWP on health status and health predictors in people with CWP.

The aim was to study the impact of CWP on health status in women and men aged 35–54 years in a sample of the general population. The aim was further to investigate lifestyle-related predictors of better health status in those with CWP in a 12- and 21-year perspective.

## Method

### Design and subjects

The present study was a part of a 21-year prospective population-based cohort study, the EPIPAIN study [28]. Participants of the EPIPAIN study constituted a representative sample (*n* = 3928) from the general population, from two municipalities in the southwest of Sweden. Every eighteenth man and woman aged 20–74 years was selected from the computerised Swedish national population register [28]. Of these, all participants aged 35–54 years in 1995 were included in the present study.

**Fig. 1:**
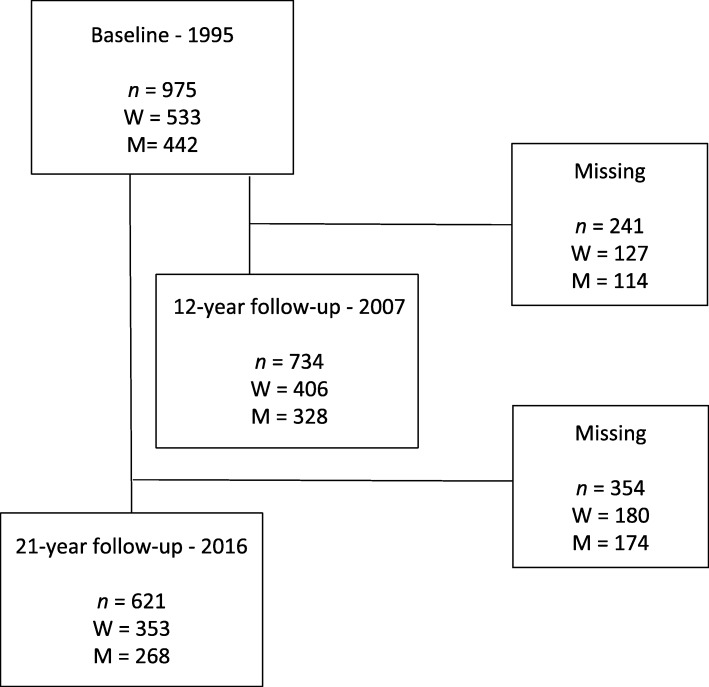
Flow-chart of participation in the study. M, men; W, women

Baseline data at 1995 included 975 participants (men/women *n =* 442/533) with a 12-year follow-up (*n* = 734, 328/406) and a 21-year follow-up (*n* = 622, 268/354) (Fig. 1). At the 21-year follow-up, the response rate was 64%. The non-responders (*n* = 354) were younger (44.5 ± 5.7 years) than the responders (45.3 ± 5.7 years, *p* = 0.05), there were no difference in gender (*p* = 0.071), and a higher proportion of people with CWP were non-responders (*p* = 0.002). Why former participants decided not to respond in the follow-ups is not known. Data were collected via a postal questionnaire over the 21 years, with follow-ups in 1998, 2003, 2007, and 2016. The data from 1998 and 2003 were not used in this study. In 2016, a web-based questionnaire was available, and two reminders were sent out on each occasion. The study followed the Strengthening the Reporting of Observational Studies in Epidemiology (STROBE) guidelines [29].

### Questionnaire

The questionnaire consisted of the standard version of the Short-Form General Health Survey (SF-36) [30] and questions about socio-demographic, pain, and lifestyle factors [28]. The Swedish validated standard version of the SF-36 and interpretation manual was used [31]. The questionnaire has 36 items, 35 of which are grouped in eight different health concepts: Physical Functioning (PF), Role function – Physical aspect (RP), Bodily Pain (BP), General Health perception (GH), Vitality (VT), Social Functioning (SF), Role function – Emotion aspect (RE), and Mental Health (MH). Each health concept has a scoring from 0 to 100 where a higher score indicates a better health status [30, 31]. Questions about pain used a pain mannequin with 18 predefined regions to localise pain regions and to determine whether the duration of pain was more than three months. Lifestyle factors measured were personal support, circle of friends, smoking, alcohol intake, physical activity, and sleeping habits [28]. The questions remained the same on each occasion, except questions regarding alcohol intake and physical activity, which were updated in 2007―and physical activity once again in 2016 according to the standards of the Swedish National Board of Health and Welfare based on Haskell et al. [32].

### Definitions

Chronic pain was defined as pain that persisted or recurred for more than 3 months [2]. Furthermore, CWP was defined according to the ACR 1990 criteria for fibromyalgia, as pain lasting for a minimum of 3 months, present on both sides of the body, below and above the waist, and in the axial skeleton [10]. An introductory key question in the questionnaire “*Have you experienced pain lasting more than three months during the last 12 months?*” identified cases with chronic pain. The key question together with the pain mannequin was the basis of the three pain categories used in this study: CWP, chronic regional pain (CRP) if the criterion for CWP was not met, and no chronic pain (NCP).

### Ethics

Written informed consent was obtained from all participants and the study adhered to the Helsinki Declaration [33]. It was approved by the Regional Ethical Review Board, Faculty of Medicine, University of Lund, Sweden (Dnr LU 389–94; Dnr 2016/132).

### Statistical analysis

Descriptive statistics represented baseline variables in 1995, including age, gender, education level, social support, circle of friends, smoking habits, alcohol intake, physical activity, sleeping habits, the eight subscales of SF-36, and the different pain groups NCP, CRP, and CWP. To test differences between groups, the two-tailed independent-samples t-test was used for continuous variables, and the χ2 test (chi-square test) was used for proportions. Gender differences in transitions to and from two different pain groups (NCP/CRP and CWP) during the 12- and 21-year follow-up were analysed using logistic regression analysis, presented as odds ratios (ORs) and 95% confidence intervals (CIs) and controlled for age. Differences in the impact of CWP on health status were measured using the SF-36 and analysed using the Swedish manual [31], and mean scores in the eight sub-subscales of the SF-36 at baseline and at the follow-ups were compared using two-tailed independent-samples t-test. Gender differences were analysed within and between the NCP/CRP and CWP groups.

Logistic regression analyses were used to study possible predictors of better health status, as measured by the SF-36 in those with CWP at baseline. The dependent variables were three subscales from the SF-36―PF, VT, and MH—which were dichotomised on the best tertiary score versus all others. Independent possible predictors were lifestyle factors (personal support, circle of friends, smoking, alcohol intake, physical activity, and sleeping habits) controlled for age and gender. Due to the small number of the outcome CWP, controlling for more confounders by multivariable-adjusted regression analysis was not possible. The three categories were chosen as primary outcomes for being of most relevance and representative of different aspects of health status [34]. The predictors are presented with their respective ORs and 95% CIs. Results were considered to be statistically significant if *p* < 0.05. The sample size used would permit detection of differences of ten points in the SF-36 with a power of at least 80% in the cross-sectional analysis, based on the minimal clinically relevant differences, calculated by multiplying the standard deviation at baseline by 0.5 [35]. Missing data has not been replaced or analysed further. The analyses were performed using the IBM SPSS 25 statistical package for Macintosh (released 2017; IBM Corp., Armonk, NY, USA).

## Results

Altogether, 975 subjects were included in the study and of these, 954 subjects fully responded to the pain questions (21 missing) and were categorised into two groups depending on pain status at baseline: NCP/CRP (*n* = 831) and CWP (*n* = 123). In the follow-up 12 years later (2007), 713 subjects fully responded (21 missing): NCP/CRP (*n* = 573)*,* and CWP (*n* = 140). In the 21-year follow-up in 2016, 621 subjects participated: NCP/CRP (*n* = 507)*,* and CWP (*n* = 114)*.* In the whole sample, there were significant gender differences in the baseline characteristics regarding education level, pain group, alcohol intake, and physical activity, and close to significant differences regarding sleeping problems. These differences were not seen in the CWP group, but there was a significant difference in having a circle of friends, which was more common in women. The baseline characteristics are presented in Table 1.

**Table 1:** Descriptive statistics for the whole sample and the CWP group at baseline in 1995

	All *n* = 975	Men *n* = 442	Women *n* = 533	*p*-value gender difference	All CWP *n* = 123	Men *n* = 33	Women *n* = 90	*p-*value gender difference
Age, years (1995)				0.981				0.602
Mean (sd)	44 (6)	45 (6)	48 (6)		47 (5)	48 (5)	46 (6)	
Education^a^, n (%)				0.010				0.894
No more than 2 years	548 (56.4)	246 (55.9)	302 (56.9)		87 (71.3)	23 (69.7)	64 (71.9)	
> 2 years	121 (12.5)	71 (16.1)	50 (9.4)		10 (8.2)	3 (9.1)	7 (7.9)	
University	222 (22.9)	91 (20.7)	131 (24.7)		15 (12.3)	5 (15.2)	10 (11.2)	
Other	80 (8.2)	31 (7.3)	48 (9.0)		10 (8.2)	2 (6.1)	8 (9.0)	
Personal support, n (%)				0.773				0.287
Yes	806 (82.9)	364 (82.5)	442 (83.2)		94 (76.4)	23 (69.7)	71 (78.9)	
No	166 (17.1)	77 (17.5)	89 (16.8)		29 (23.6)	10 (30.3)	19 (21.1)	
Circle of friends, n (%)				0.380				0.026
Yes	615 (63.5)	273 (62)	342 (64.8)		64 (52.9)	12 (36.4)	52 (59.1)	
No	353 (36.5)	167 (38)	186 (35.2)		57 (47.1)	21 (63.6)	36 (40.9)	
Pain group, n (%)				0.000				
NCP	593 (62.2)	289 (67.1)	304 (58.1)					
CRP	238 (24.9)	109 (25.3)	129 (24.7)					
CWP	123 (12.9)	33 (7.7)	90 (17.2)					
Smoking habits, n (%)				0.325				0.918
No, never smoked	452 (46.5)	194 (44)	258 (48.6)		55 (44.7)	15 (45.5)	40 (44.4)	
No, have stopped	281 (28.9)	136 (30.8)	145 (27.3)		33 (26.8)	8 (24.2)	25 (27.8)	
Yes, smoker	239 (24.6)	111 (25.2)	128 (24.1)		35 (28.5)	10 (30.3)	25 (27.8)	
Alcohol intake, n (%)				0.000				0.125
Never/almost never	313 (32.2)	96 (21.9)	217 (40.8)		70 (56.9)	17 (51.5)	53 (58.9)	
Every month	407 (41.9)	198 (45.1)	209 (39.3)		36 (29.3)	8 (24.2)	28 (31.1)	
Every week	246 (25.3)	143 (32.6)	103 (19.4)		17 (13.8)	8 (24.2)	9 (10.0)	
> 4 times a week	5 (0.5)	2 (0.5)	3 (0.6)		0 (0)	0 (0)	0 (0)	
Physical activity^b^, n (%)				0.002				0.582
Never	463 (28)	237 (54.2)	226 (42.9)		54 (43.9)	17 (51.5)	37 (41.1)	
Moderate	337 (35)	134 (30.7)	203 (38.5)		42 (34.1)	10 (30.3)	32 (35.6)	
Active	164 (17)	66 (15.1)	98 (18.6)		27 (22)	6 (18.2)	21 (23.3)	
Sleeping problems^c^, n (%)				0.053				0.731
No	495 (52.9)	240 (56.3)	255 (50)		20 (16.8)	6 (18.8)	14 (16.1)	
Yes	441 (47.1)	186 (43.7)	255 (50)		99 (83.2)	26 (81.3)	73 (83.9)	

^a^ Highest finished education: *No more than 2 years* = after elementary school; *> 2 years* = more than 2 years after elementary school; *University* = education at university level; *Other* = all others

^b^ *Never* = < 30 min/day; *Moderate* = 1–2 times/week or 30–90 min/week; *Active* = > 2 times/week or > 120 min/week

^c^ *No* = no or minor sleeping problems; *Yes* = moderate to severe sleeping problems

**Fig. 2:**
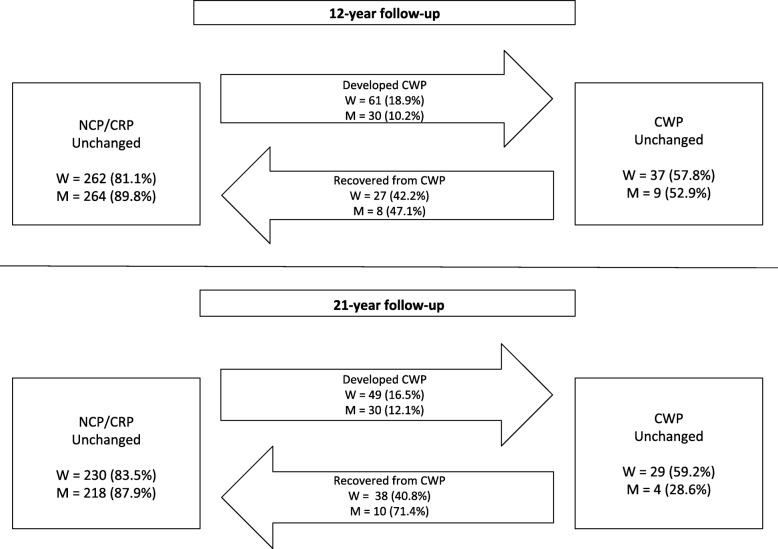
Transitions between the two different pain groups during the 12- and 21-year follow-up, n (%); W, woman; M, man

At baseline, more women than men reported having CWP and a higher proportion of women than men remained in the CWP group at the 12- and 21-year follow-up (Fig. 2). Of those with NCP/CRP at baseline, a higher proportion of women than men developed CWP at the 12-year follow-up (OR 2.04; CI 1.27–3.26; *p* = 0.003). In women and men with CWP at baseline, no significant difference was found regarding recovery from CWP (OR 1.22; CI 0.42–357; *p* = 0.717). At the 21-year follow-up, no significant gender difference was found regarding the development of CWP (OR 1.43; CI 0.88–2.35; *p* = 0.151) but men recovered from CWP to a greater extent than women (OR 3.79; CI 1.00–14.33; *p* = 0.050).

### Impact of CWP on health status

**Fig. 3:**
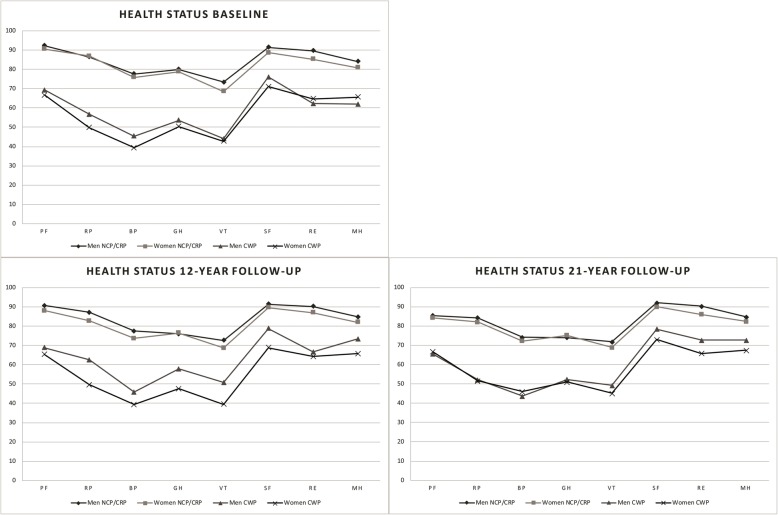
Results from the cross-sectional analysis of health status in the NCP/CRP and CWP groups, men and women separate, presenting the eight different SF-36 sub-subscales at baseline and at the 12- and 21-year follow-up. The SF-36 sub-scales are: physical function (PF); role function – physical aspect (RP); bodily pain (BP); general health perception (GH); vitality (VT), social functioning (SF); role function – emotional aspect (RE), and mental health (MH)

People with CWP reported having a lower health status in all eight SF-36 sub-subscales compared to the NCP/CRP group (*p* < 0.001) (Fig. 3). Within each of the two pain groups, there were no gender differences regarding health status at baseline, nor at the 21-year follow-up. At the 12-year follow-up, there were statistically significant gender differences in the CWP group regarding BP (mean difference *δ* = 6.54; *p* = 0.049), GH (*δ* = 10.26; *p* = 0.015), VT (*δ* = 11.20; *p* = 0.008), and SF (*δ* = 10.0; *p* = 0.036), with women reporting worse scores.

### Predictors of health

Altogether, 83 subjects (3 missing due to incomplete questionnaire) with CWP at baseline answered the questionnaire at the 12-year follow-up and were included in the logistic regression analysis. For the 21-year follow-up, 63 subjects were included (5 missing due to incomplete questionnaire). All predictors were controlled for age and gender, except for age (which was controlled for gender) and gender (which was controlled for age). Male gender was predictive of better health status regarding VT (OR 3.03; CI 1.02–9.00) at the 12-year follow-up and almost significant for PF (OR 2.91; CI 0.98–8.26) but not for MH. Being a previous smoker was associated with better PF (OR 5.21; CI 1.12–23.03) and VT (OR 4.38; CI 1.04–18.44) compared to being a current smoker. Personal support was associated with better VT (OR 9.27; CI 1.02–54.47), and having no sleeping problems was predictive of having a better health status regarding VT (OR 4.76; CI 1.38–16.46) and MH (OR 3.48; CI 1.07–11.34) (Table 2).

**Table 2:** Health predictors at the 12-year follow-up for people with CWP at baseline in 1995, *n* = 83

Predictors	Physical Functioning (PF)		Vitality (VT)		Mental Health (MH)	
	OR (95% CI)	*p*	OR (95% CI)	*p*	OR (95% CI)	*p*
Age	0.96 (0.88–1.05)	0.415	0.93 (0.86–1.02)	0.114	1.00 (0.91–1.09)	0.902
Gender						
Woman	1		1		1	
Man	2.91 (0.98–8.62)	0.053	3.03 (1.02–9.00)	0.046	1.67 (0.56–4.98)	0.360
Personal support						
No	1		1		1	
Yes	1.98 (0.52–7.51)	0.317	9.27 (1.58–54.47)	0.014	2.48 (0.61–10.07)	0.204
Circle of friends						
No	1		1		1	
Yes	2.21 (0.76–6.43)	0.148	0.28 (0.64–4.89)	0.276	1.09 (0.40–2.95)	0.862
Smoking						
Yes	1		1		1	
No, never	1.44 (0.38–5.50)	0.598	1.62 (0.46–5.72)	0.456	1.82 (0.50–6.63)	0.363
No, have stopped	5.21 (1.12–23.03)	0.030	4.38 (1.04–18.44)	0.044	2.36 (0.56–10.05)	0.244
Alcohol intake						
Never/rarely	1		1		1	
Every month	1.37 (0.47–4.01)	0.569	1.26 (0.44–3.59)	0.666	1.25 (0.44–3.57)	0.675
Every week	2.01 (0.53–7.69)	0.309	2.17 (0.57–8.28)	0.255	1.02 (0.25–4.09)	0.982
> 4 times/week^a^	–		–		–	
Physical activity^b^						
Never	1		1		1	
Moderate	0.57 (0.19–1.72)	0.316	0.87 (0.30–2.56)	0.802	0.65 (0.21–2.02)	0.453
Active	1.27 (0.35–4.62)	0.716	1.67 (0.46–6.03)	0.434	2.49 (0.70–8.83)	0.158
Sleeping problems^c^						
Yes	1		1		1	
No	2.22 (0.68–7.28)	0.187	4.76 (1.38–16.46)	0.014	3.48 (1.07–11.34)	0.038

^a^ There was no-one with CWP in this group and “every week” became the reference

^b^ *Never* = < 30 min/day; *Moderate* = 1–2 times/week or 30–90 min/week; *Active* = > 2 times/week or > 120 min/week

^c^ *Yes* = moderate to severe sleeping problems; *No* = no or minor sleeping problems. Controlled for age and gender, except for age (which was controlled for gender) and gender (which was controlled for age)

For the 21-year follow-up, male gender was predictive of better health status for PF (OR 6.76; CI 1.82–25.13) and VT (OR 5.71; CI 1.53–21.34). Being a previous smoker was found to be a predictor of better PF (OR 7.83; CI 1.05–58.46) and also weekly intake of alcohol (OR 4.94; CI 1.05–23.33) in the 21-year follow-up. Having no sleeping problems was found to be a predictor of better MH (OR 4.45; CI 1.05–18.80) (Table 3).

**Table 3:** Health predictors at the 21-year follow-up for people with CWP at baseline in 1995, *n* = 63

Predictors	Physical Functioning (PF)		Vitality (VT)		Mental Health (MH)	
	OR (95% CI)	*p*	OR (95% CI)	*p*	OR (95% CI)	*p*
Age	0.93 (0.84–1.04)	0.201	1.05 (0.95–1.17)	0.310	1.06 (0.96–1.17)	0.284
Gender						
Woman	1		1		1	
Man	6.76 (1.82–25.13)	0.004	5.71 (1.53–21.34)	0.010	3.35 (0.92–11.54)	0.056
Personal support						
No	1		1		1	
Yes	1.19 (0.26–5.41)	0.825	2.01 (0.47–9.26)	0.333	2.40 (0.52–11.00)	0.260
Circle of friends						
No	1		1		1	
Yes	0.47 (0.14–1.56)	0.214	1.41 (0.44–4.51)	0.562	0.89 (0.29–2.73)	0.839
Smoking						
Yes	1		1		1	
No, never	4.23 (0.65–27.56)	0.131	0.67 (0.15–3.02)	0.602	0.99 (0.22–4.39)	0.990
No, have stopped	7.83 (1.05–58.46)	0.045	1.50 (0.31–7.38)	0.616	1.32 (0.27–6.52)	0.736
Alcohol intake						
Never/rarely	1		1		1	
Every month	2.55 (0.62–10.45)	0.193	1.40 (0.40–4.93)	0.603	0.55 (0.15–2.03)	0.367
Every week	4.94 (1.05–23.33)	0.044	1.39 (0.34–5.79)	0.647	0.88 (0.22–3.59)	0.862
> 4 times/week^a^	–		–		–	
Physical activity^b^						
Never	1		1		1	
Moderate	0.77 (0.20–2.97)	0.698	1.02 (0.27–3.82)	0.977	2.96 (0.70–12.48)	0.139
Active	0.35 (0.07–1.82)	0.212	0.87 (0.21–3.60)	0.848	3.05 (0.66–14.06)	0.154
Sleeping problems^c^						
Yes	1		1		1	
No	2.01 (0.47–8.63)	0.346	3.45 (0.80–14.85)	0.096	4.45 (1.05–18.80)	0.042

^a^ There was no-one with CWP in this group and “every week” became the reference

^b^ *Never* = < 30 min/day; *Moderate* = 1–2 times/week or 30–90 min/week; *Active* = > 2 times/week or > 120 min/week

^c^ *Yes* = moderate to severe sleeping problems; *No* = no or minor sleeping problems. Controlled for age and gender, except for age (which was controlled for gender) and gender (which was controlled for age)

## Discussion

The aim of this study was to investigate the impact of CWP on health status in women and men, and to investigate lifestyle predictors of better health status in those with CWP at baseline, with a 12- and 21-year follow-up. The results showed a more than double the prevalence of CWP in women than in men, a result that is in line with previous research [11, 12].

During the 12-year follow-up, women developed CWP to a greater extent than men, but no gender difference was found in recovery from CWP. During the 21-year follow-up, the results were the opposite and women did not develop CWP more than men but recovered to a lesser extent from CWP than men. Previous studies have shown contradictory results over a shorter period of time (3 and 7 years) where women with NCP or CRP did not develop CWP or persistent CWP more than men [36, 37]. However, an 11-year follow-up found that men had half of the odds of having persistent CWP compared to women [16]. This could indicate that men have CWP for a shorter time than 21 years, and that they recover to a greater extent than women some time after 7–12 years of persistent CWP. Why women would have more prolonged of CWP is not known, but this has been found in other studies [16, 20, 37].

The present study showed that both women and men with CWP had reduced health status compared to the NCP/CRP group, which corroborates the results of previous studies [7, 9, 34, 38]. There were no gender differences in health status at baseline or at the 21-year follow-up in those with CWP, indicating that men and women with CWP have the same worsened health status. During the 12-year follow-up, there were some gender differences in health status, however, only the vitality (VT) result was clinically relevant. This could be explained by the fact that the individuals moved between the different pain groups in the different time points.

Most previous studies have examined the impact that chronic pain has on health status, and not specifically the condition CWP when comparing the genders [3, 6, 28, 36, 39]. This study is one of the first to focus on gender differences regarding the effect that CWP has on health status. However, one previous study found that despite the fact that women reported having more pain locations than men (CWP was more frequent in women and CRP in men), the women reported being more satisfied with their social life and with life in general than men, and men had lower self-reported mental health than women [26]. Gender differences in health status when studying chronic pain vary [4, 25, 26], highlighting the importance to separate different types of pain when studying health status and gender differences. The higher prevalence of CWP in women could be one explanation as to why some studies have reported a worse health status for women than for men, and that men handle pain better when studying only chronic pain and not taking the presence of CWP into account.

In the present study, male gender was found the be a predictor of better VT in both the 12-year and the 21-year follow-up, and a predictor of better physical function (PF) in the 21-year follow-up. Male gender has been reported to be a predictor of health in previous studies [6, 16]. Why male gender would be such a predictor is not fully known, but explanations put forward have been, for example, hormonal factors [23]—and that women tend to be more willing to report pain than men [20].

Having personal support was a decisive health factor regarding VT in the 12-year follow-up. No previous studies have investigated the association between personal support and health status in the long-term when living with CWP. However, another study from the EPIPAIN cohort with the larger sample found a similar result with an eight-year follow-up, where personal support was associated with increased health status in VT and mental health (MH) in those with chronic pain at baseline [6]. The lack of research on the association between social support and health status together with the results of the present study indicates that more research will be required to understand this possibly beneficial association.

During the 12-year follow-up, being a former smoker was a predictor of better health regarding PF and VT, compared to being a current smoker. In the 21-year follow-up, the prediction of better health was only present for PF. It has been found that smokers report having higher pain intensity, which is in turn associated with a negative interference with life enjoyment [40]. There is a dose-response relationship between smoking on the one hand and pain intensity and duration on the other when having chronic pain [15], and reversibility of pain intensity in the long-term is presumed when stopping smoking [15, 41]. Since giving up smoking improves the physical health [42], it could explain the result of better PF in former smokers, and would also explain why non-smokers do not show any change in PF. However, the study has not taken into consideration *when* the participants stopped smoking or numbers of years smoking, so no further conclusions can be drawn.

Consumption of alcohol every week was found to be a health predictor regarding PF in the 21-year follow-up. Previous research has shown that low to moderate alcohol intake is associated with a better quality of life in people with fibromyalgia [43]. Another study found that consuming alcohol more than eight times a month had a protective factor against the persistence of CWP [16]. Moreover, moderate intake of alcohol has been shown to reduce pain severity, and it has been suggested that the pain-relieving effects of alcohol improve the physical function when having chronic pain [44], which could be one explanation for the current study results. Furthermore, the association found could also be due to positive social factors linked to alcohol consumption [45] and/or that people with illnesses refrain from drinking alcohol [46]. However, the quantity consumed was not taken into consideration in the present study, and therefore no further conclusions can be made.

Having no sleeping problems was found to be a predictor of better health regarding VT and MH in the 12-year follow-up, compared to having sleeping problems. In the 21-year follow-up, having no sleeping problems was associated with better MH. These results suggest that sleep is a critical factor for improvement of mental health when living with long-term CWP. Improving restorative sleep is associated with the resolution of CWP where less pain is reported [19], which in turn gives better health [7]. In previous studies, sleeping problems have also been reported as a predictor of the onset of CWP [18] and the persistence of CWP [16]. It is therefore a challenge to find out whether it is the pain that causes sleeping problems and therefore worse health, or whether the sleeping problems cause the pain and therefore worse health.

Two major strengths of the present study were that the study was based on a larger cohort and therefore had a large sample size with a long-term follow-up of 21-years. The age range was chosen because it represents mid-life, and is the time in life before and during which chronic pain becomes more prevalent (at 40–50 years) [8, 12, 21]. This was therefore considered a strength. The study used an established questionnaire to measure health status [30, 31], and the other questions had been tested and resulted in good content, criterion, and face validity as well as test-retest reliability [47]. The study only examined pain according to pain location and duration, which can be considered to be both a strength (since CWP seems to be an important subgroup of chronic pain) and a limitation (since pain intensity was not part of the analysis). Another limitation could be that it due to sample size was not possible to control for all possible confounding factors. The long follow-up time, although being a strength, might also decrease the causality between baseline predictors and outcome. Compared to the responders, the group of non-responders had a higher proportion of people with CWP which is considered a limitation and could affect the generalisability. However, with the large sample size and no or minimal differences in non-responders regarding gender and age it is still considered possible to generalise the results to men and women with CWP in mid-life.

## Conclusions

The study showed that more women than men had persistent CWP in a 12- and 21-year time frame, but when suffering from CWP, health status was equally as bad in women and men. This suggests that even though men have a lower prevalence of CWP, the condition should be regarded as having the same impact on men and women in health care. Modifiable health factors such as having no sleeping problems were strongly associated with better mental health, and personal support may be of importance for better vitality. The results state that the persistence of CWP differs more between genders after 7 to 12 years, when men recover to a greater extent than women. Further research should continue studying the persistence of CWP and possible confounders, to establish the gender-related differences in the long-term.

## Data Availability

The datasets used and analysed during the current study are available from the corresponding author on reasonable request.

## Abbreviations

BP: Bodily Pain
CI: Confidence interval
CRP: Chronic regional pain
CWP: Chronic widespread pain
GH: General Health
MH: Mental Health
NCP: No chronic pain
OR: Odds ratio
PF: Physical Functioning
RE: Role function – Emotional
RP: Role function – Physical
SF: Social Functioning
VT: Vitality
